# Virucidal activity of *Garcinia parvifolia* leaf extracts in animal cell culture

**DOI:** 10.1186/s12906-019-2586-5

**Published:** 2019-07-10

**Authors:** Aziera Adnan, Zeenathul Nazariah Allaudin, Homayoun Hani, Hwei-San Loh, Teng-Jin Khoo, Kang Nee Ting, Rasedee Abdullah

**Affiliations:** 1 Department of Veterinary Pathology and Microbiology, Faculty of Veterinary Medicine, Universiti Putra Malaysia, 43400 Serdang, Selangor Malaysia; 2 Department of Cell Biology and Physiology, School of Medicine, University North Carolina, Chapel Hill, NC 27599 USA; 3 School of Biosciences, University of Nottingham Malaysia, Jalan Broga, 43500 Semenyih, Selangor Malaysia; 4 School of Pharmacy, University of Nottingham Malaysia, Jalan Broga, 43500 Semenyih, Selangor Malaysia; 5 Department of Veterinary Laboratory Diagnostics, Faculty of Veterinary Medicine, Universiti Putra Malaysia, 43400 Serdang, Selangor Malaysia

**Keywords:** Pseudorabies virus, Ethyl acetate, Ethanol, Hexane, Plaque reduction assay, Cytopathic effect reduction assay, Inhibition assay, Virucidal assay, Selectivity index

## Abstract

**Background:**

*Garcinia* species contain bioactive compounds such as flavonoids, xanthones, triterpernoids, and benzophenones with antibacterial, antifungal, anti-inflammatory, and antioxidant activities. In addition, many of these compounds show interesting biological properties such as anti-human immunodeficiency virus activity. *Garcinia parvifolia* is used in traditional medicine. Currently, the antiviral activity of *G. parvifolia* is not known.

**Methods:**

This study was conducted to determine the effects of ethyl acetate (45 L Ea), ethanol (45 L Et), and hexane (45 L H) leaf extracts of *G. parvifolia* on the infectivity of pseudorabies virus (PrV) in Vero cells. The antiviral effects of the extracts were determined by cytopathic effect (CPE), inhibition, attachment, and virucidal assays.

**Results:**

The 50% cytotoxicity concentration (CC_50_) values obtained were 237.5, 555.0, and < 1.25 μg/mL for 45 L Ea, 45 L Et, and 45 L H, respectively. The 45 L Ea showed the greatest viral inhibition potency of 75% at 125 μg/mL. Both 45 L Ea and 45 l Et caused 100% residual viral inhibition at 250 μg/mL. The selectivity index values for 45 L Ea, 45 L Et, and 45 L H were 2.65, 1.75, and 0.10 showing that 45 L Ea had the greatest antiviral activity among the three extracts.

**Conclusion:**

This study showed that ethyl acetate is the best solvent to be used to obtain extract from *G. parvifolia* leaves with potent antiviral activities.

## Background

There are several ways by which therapeutic compounds interfere with viral replication. The antiviral effects can either be through prevention of viral attachment to host cell, binding to enzymes responsible for transcription, and prevention of cleavage of viral particles [1]. Viruses mutate over time and develop resistance to antiviral drugs and therapeutic compounds [2]. Thus, there is a need to discover and develop antiviral agents that do not become ineffective over time owing to development of resistance by the virus. But the pipeline of new drugs is drying up. There would be a tremendous benefit by integrating combinations of modern drugs with traditional medicinal plant extracts that have been used as folk medicine to broaden the curing spectrum via generating synergistic effects.

Traditional medicinal trees are evergreen, abundant and available year round in tropical regions. Local communities used various parts of these trees in their traditional practice because of their high nutritive values but yet some of their detailed medicinal properties remain unknown. The plant studied, *Garcinia parvifolia* produces cherry-like fruit which is locally known as “asam kandis” or “asam kundong” [3], whilst the young leaves are sometimes eaten as a vegetable. The leaf extracts of this plant were screened against pseudorabies virus (PrV). It is a broad host range herpesvirus, causes fatal encephalitis in a wide variety of animal species except its natural host, the adult pig [4–7]. Since PrV is not a human pathogen, it is safe to be used in a laboratory set-up. The virus can easily be grown in the laboratory thus it is practical and convenient to be used in the screening and development of antiviral drugs or compounds.

*G. parvifolia* which belongs to the family of Clusiaceae (Guttiferae), is native in tropical and subtropical countries of South East Asia such as here in Malaysia, Thailand, Brunei, and Indonesia [8, 9]. Garcinia is known to produce xanthones and benzophenones [9, 10] and many of these compounds show interesting biological activities including anti-human immunodeficiency virus activity [9, 10]. There are at least 300 distinct *Garcinia* species and many contains bioactive compounds to include flavonoids, xanthones, triterpernoids, and benzophenones with beneficial biological activities [11–14]. The crude extracts of some parts of *G. parvifolia* have shown antiplasmodial, antioxidant, cytotoxic and antibacterial activities [15]. However, the antiviral properties of the *G. parvifolia* extract are not known. Since *G. parvifolia* has rather similar properties with other *Garcinia sp*, it potentially has antiviral activities and hence is of great interest to test in the current study. In this study, their leaf extracts were obtained by using either ethyl acetate, ethanol, or hexane and screened for the efficiency to inhibit PRV.

## Methods

### Plant collection and crude plant extracts

Approval for *Garcinia parvifolia* plant collection was obtained from Forestry Department Peninsular Malaysia*. G. parvifolia* leaf samples were obtained from the Sungai Congkak Recreational Forest Hulu Langat, Selangor, Malaysia (3.209675°N, 101.844327°E) and further authenticated by Forest Research Institute Malaysia (FRIM) botanist service of Richard Chung Cheng-Kong. The herbarium voucher specimens (herbarium code: UNMC45) were deposited at the Herbariums of the Faculty of Science, University of Nottingham Malaysia and FRIM. The ground plant leaves were subjected to sequential extraction procedures as described previously [16, 17]. Briefly, crude plant extracts prepared from harvested leaves, using hexane and ethanol (RCI Labscan, Thailand) and ethyl acetate (R&M Chemicals, Malaysia) solvents, that marked 45 L H, 45 L Et, and 45 L Ea, respectively were provided by Dr. Teng Jin Khoo, the University of Nottingham, Malaysia following the extraction steps. Leaves of *G. parvifolia* were washed with sterile deionized water, shredded into small pieces, and let them dried in a closed room at 25–28 °C for 2 weeks. The dried leaves were milled, weighed and soaked in 95% ethanol at a fraction of 1:8 (fragment: ethanol) at room temperature for 24 h. The leaf ingredients were then sequentially extracted according to solvent polarity of ethanol, followed by hexane and then ethyl acetate. Each plant extract was saturated three times before undergoing the reduced pressure rotary evaporation processing at 40 °C. The concentrated crude extracts were kept at − 20 °C [17].

### Preliminary phytochemical analysis

Several phytochemical tests were performed to confirm the presence of secondary metabolites: saponin, flavonoid, tannin, phenolic content, steroid and terpenoid. Approximately 12.5 mg of each leaf extract from the *G. parvifolia* plant (45 L H, 45 L Et, and 45 L Ea) was used.

#### Test for saponin: frothing method

Each extract was dissolved in 5 mL of purified water in the test tube covered with cork according to method established by Ibrahim and Ibrahim [18]. The samples were sonicated for 15 min at 40 °C. Samples that had particles were filtered before vigorously shaken for 30 s and left for another 45 min. Persistence frothing formation showed the positive results for saponin [18].

#### Test for flavonoid

The flavonoid analysis was prepared following the Modified Shinoda Test [19]. Each extract was added with 5 mL of dimethyl sulfoxide (DMSO; Merck, Germany), next with 3–4 cm of magnesium turnings and 6 drops of 36% concentrated HCl. Various colours like orange, pink, red to purple represent different flavonoids like flavones, flavonols, 2,3-dihydro derivates and xanthone, respectively.

#### Test for tannin and phenolic content

This test established by Mojab and colleagues [20] uses two reagents which are gelatin for protein precipitation and ferric chloride to confirm the presence of phenolic compound. Each extract was dissolved in 5 mL purified water and sonicated for 15 min at 40 °C. The volume was divided into three portions: one for control, the remaining for gelatin precipitation and phenolic content analysis. All portions were then added with 6 drops of 1% ferric chloride. White fog or precipitation with 5 mL of 1% gelatin, while brownish-green or blackish-blue colour shows the presence of phenolic compounds [20].

#### Test for steroid and terpenoid

Both steroid and terpenoid tests were accomplished using Salkowski Test [21, 22]. Every extract was dissolved in 5 mL of DMSO and sonicated for 15 min at 40 °C. One milliliter of solution was added with 1 mL chloroform and equal volume of concentrated sulphuric acid slowly at the side of the test tube. Upper layer exhibited red colour and sulphuric layer showed yellow colour with green fluorescence. These two layers observed and reddish-brown at the interface corresponds to the presence of terpenoid while the blue or green interface indicates steroid compound.

### Pseudorabies virus (PrV)

The stock PrV strain amorphous inclusion protein (AIP) used in this study was an established virus at the Virology Laboratory, Faculty of Veterinary Medicine, Universiti Putra Malaysia. Quantitation of stock virus was conducted on Vero cells (ATCC No. CCL-81) by using the plaque-forming assay. The virus was stored at − 80 °C.

### Vero cell culture

Vero cells were grown and maintained in RPMI 1640 supplemented with 10% fetal bovine serum (FBS), 1% Penicillin (100 U/mL), 1% Streptomycin (100 mg/mL), and 1% Fungizone (2.5 mg/mL). The cells were seeded into sterile 96-well and 24-well flat bottom plates. Vero cells were incubated under 5% CO_2_ humidified atmosphere at 37 °C.

### Plant extract

The plant extracts were dissolved in pure DMSO and diluted with sterile de-ionized water to a total volume of 1 mL. The stock solution was dissolved for 48 h in either 10 or 50% DMSO to obtain 10 and 50 mg/mL extract concentrations, respectively and stored at − 20 °C.

### 50% tissue culture infectious dose (TCID_50_)

Vero cells were seeded into flat-bottom 96-well microtiter plates at 2 × 10^4^ cells/well and incubated for 24 h under 5% CO_2_ humidified atmosphere at 37 °C. Serial dilution of virus stock (10^8^ PFU/ml) was prepared in media with FBS. So the working stock was formed forth dilution that was 1 × 10^4^ PFU/ml, then incubated with the Vero cells for 72 h. The cytopathic effect (CPE) and proportional distance (PD) were calculated using the following formula [23]; PD = (% of wells infected at dilution > 50%) - (50% infection) / (% of wells infected at dilution > 50%) – (% of wells infected at dilutions < 50%). The tissue culture infectious dose 50% (TCID_50_) is calculated by using the following formula: TCID_50_ = 10^log total dilution > 50% - (1 × log h)^.

### Pseudorabies virus quantification

Vero cells (1.6 × 10^5^ cells) in RPMI 1640 containing 2% FBS were seeded in each well of a 24-well plate and incubated under 5% CO_2_ humidified atmosphere at 37 °C in for 24 h. The medium was discarded, replaced with fresh medium and plates were again incubated under 5% CO_2_ humidified atmosphere at 37 °C for 48 h. The virus was diluted with fresh RPMI 1640 with 2% FBS to obtain a working virus solution. One hundred microliters of virus suspension containing 1X10^7^ PFU PrV was added to each containing Vero cells in 1 mL of 1% methylcellulose and 2% FBS and the plate incubated rocking for 1 h. Infected cells were fixed with methanol and stained with 0.5% crystal violet solution for 30 min. The number of plaques formed were counted and expressed as PFU/mL.

### Cytotoxicity assay

The cytoxicity assay was conducted according to the method described by Serkedjieva and Ivancheva [24] and Malik et al. [17]. Confluent monolayer Vero cells in 96-well flat-bottom plated were treated with *G. parvifolia* leaf extracts at 62.5, 125, 250, 500 and 1000 μg/mL. Vero cells treated with various concentrations of DMSO (100 μL of either 0.078, 0.157, 0.3, 0.625, 1.25, 2.5, 5 or 10% in PBS) served as positive controls. However, the final concentration of DMSO in the working concentration was less than 0.3%. Nontreated Vero cells served as the negative control. Positive controls were treated with DMSO at the same concentrations as the plant extracts.

The plates were incubated under 5% CO_2_ humidified atmosphere at 37 °C for 48 h. Twenty microliters of 0.5 mg/mL MTT solution was added to each well and incubated under 5% CO_2_ humidified atmosphere at 37 °C in for 4 h. The incubation solution was removed and replaced with 100 μL of pure DMSO. The plates were shaken for 15 min before reading with a multiplate reader (Tecan Sunrise, Männedorf, Switzerland) at 570 nm. The reference wavelength was set at 650 nm. The 50% Cytotoxicity Concentration (CC_50_) was determined using the following formula: CC_50_ determined by (∆OD = (OD _value of treated group_ /(OD _value of medium control_) × 100) where OD_treatment_ is optical density for treatment group and OD_control_ for control. The assay was done in triplicates.

### Plaque reduction assay

The experiment was performed according to the method described by Zhu et al. [25] with brief modifications. Confluent monolayer of Vero cells grown in 24-well culture plates were treated with 100 μl of 37.5, 75, 150, and 300 μg/mL for 45 L Ea; 25, 50, 100, and 200 μg/mL for 45 L Et and 2.5, 5, 10, 20, 40 μg/mL for 45 L H. Besides, 100 PFU per 100 μL PrV was added and incubated at 37 °C for 90 min. The virus and extract mixture was discarded and 1 mL of 1% methylcellulose and 2% FBS mixture were added to the wells and the plate was incubated in 5% CO_2_ humidified atmosphere at 37 °C for 48 h. Infected cells were fixed with methanol and stained with 0.5% crystal violet solution for 30 min. The number of plaques was counted and 50% inhibition concentration (IC_50_) calculated by the following formula: IC_50_ = [1-(PFU_Treatment_/PFU_Control_)] × 100 where PFU_treatment_ = PFU of treatment and PFU_Control_ = PFU all control cells. The assay was done in quadruplicates.

### Inhibition assay

The experiment was performed according to the method described by Zhu et, al. [25]. Confluent monolayers of Vero cells grown in 24-well culture plates were infected with 100 PFU of PrV in 200 μL reaction volume and incubated under 5% CO_2_ humidified atmosphere at 37 °C for 90 min. The incubation solution was removed and 200 μL of 62.5, 125, 250, and 500 μg/mL 45 L Ea or 45 L Et; 1.25, 2.5, 5, 10, and 20 μg/mL 45 L H was added as 1 mL in 1% methyl cellulose and 2% FBS mixture. The plates were incubated at 37 °C in 5% CO_2_ humidified atmosphere for 48 h. The cells were fixed and stained with 0.5% crystal violet and the IC_50_ was calculated as described for the plaque reduction assay.

### Attachment assay

The assay was conducted according to the Logu et al., [26]. Confluent monolayer Vero cells were pre-chilled at 4 °C for 1 h. Confluent monolayers of Vero cells were infected with 50 μL of 1 × 10^2^ PFU/mL PrV in the presence of 100 μL of 6.125, 12.5, 25, 50, or 100 μg/mL 45 L Ea, 45 L Et, or 45 L H extracts in 1% DMSO. Vero cells infected with PrV but not treated with extract served as positive control while those neither infected with PrV nor treated with extract served as the negative controls. The cells were chilled at 4 °C for 3 h and the incubating mixture removed. The cells were washed trice with PBS, supplemented with RPMI 1640 containing 10% FBS, and incubated under 5% CO_2_ humidified atmosphere at 37 °C for 48 h. The cells were observed daily to determine the CPE. When 100% CPE was observed, the cells were subjected to MTT assay as described in the cytotoxicity assay. The IC_50_ of the plant extract was determined as described earlier.

### Virucidal assay

The experiment was performed according to the method described by Carlucci et. al. [27]. 1 × 10^6^ PFU PrV were mixed with 125, 250 or 500 μg/mL of 45 L Ea, 62.5, 125 or 250 μg/mL of 45 L Et, and 1.25, 2.5, 5.0 or 10 μg/mL of 45 L H and then incubated at 25 °C for 6 h. One hundred microliters of virus suspension or extract was mixed with 100 μL RPMI media containing 2% FBS and added to confluent monolayer Vero cells in a 24-well plate and incubated at 37 °C for 90 min. The incubating mixture was removed and replaced with 1 mL of 1% methyl cellulose and 2% FBS mixture and incubated under 5% CO_2_ humidified atmosphere at 37 °C for 48 h. Formed plaques were fixed with methanol and stained with 0.5% crystal violet solution for 30 min. The number of plaques was counted and residual virus infectivity was determined by the following formula: Plaque formation (PFU) = Number of plaques × (1/viral inoculation) × (1/diluted fold). The IC_50_ was calculated using the formula described in the plaque reduction assay.

### MTT assay

The 3-(4,5-dimethylthiazol-2-yl)-2,5-diphenyltetrazolium bromide (MTT) assay (Calbiochem®, Germany) was conducted when the cytotoxicity and antiviral assays give 100% CPE results. The Vero cells were added to 20 μL of MTT solution with a final concentration of 0.5 mg/mL in the 96-well plate and the plate wrapped in aluminium foil to protect from light. The Vero cells were reincubated under 5% CO_2_ humidified atmosphere at 37 °C for 4 h. The incubating solution was removed and 100 μL pure DMSO added to all wells to dissolve the formazan crystals. The plates were gently shaken for 15 min to dissolve formazan crystals. The absorbances were read with a multiplate reader [Sunrise, Tecan] at 570 nm with a reference wavelength of 650 nm. The CC_50_ was determined as described in the cytotoxic assay.

### Data analysis

Two-way ANOVA was used to determine the difference between means of experimental data followed by post/hoc test using SPSS 19.0. Differences between means were considered significant at *P* < 0.05.

## Results

### Phytochemical constituents

Six phytochemical constituents, namely saponin [18], flavonoid [19], tannin and phenolic [20] as well as terpenoid and steroid [21, 22] contents were successfully determined in 45 L extracts based on the established methods. Phytochemical analysis demonstrated that 45 L Et contained all the six constituents tested, namely the saponin, flavonoid, tannin, phenolic, terpenoid and steroid. 45 L Ea and 45 L Et had rather similar phytochemical profiles except for 45 L Ea lacked of saponin compound. 45 L H contained terpenoid and steroid only. Saponin was only found in the 45 L Et. Terpenoid and steroid were found in the three extracts.

### Cytotoxicity

**Fig. 1:**
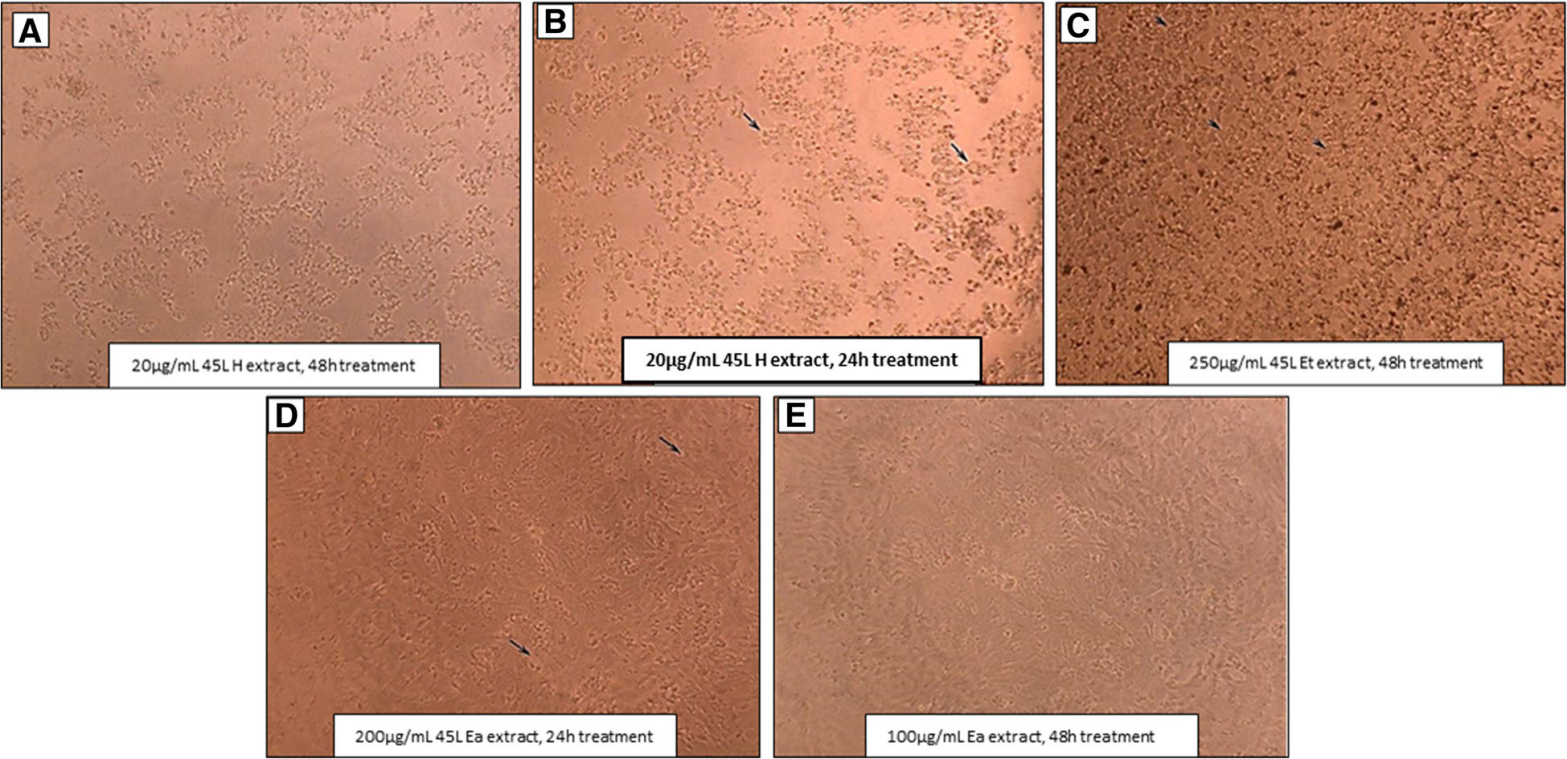
Cytotoxicity scoring of Vero cells treated with *G. parvifolia* leaf extract. **a** Score 1 – severe cytotoxicity, no visible cells (20 μg/mL 45 L H extract at 48 h), **b** Score 2 – significant cytotoxicity, a few intact cells (20 μg/mL 45 L H extract at 24 h), **c** Score 3 – Moderate cytotoxicity, altered cell morphology, cell well-spaced out (250 μg/mL 45 L Et extract at 24 h), **d** Score 4 – Mild cytotoxicity, altered cell morphology, cells closely spaced (200 μg/mL 45 L Ea extract at 24 h), **e** Score 5 – No cytotoxicity, normal cell morphology, dense cell distribution (100 μg/mL 45 L Ea extract at 48 h). (10×). 45 L H, 45 L, Et, and 45 L Ea is hexane, ethanol and ethyl acetate extracts, respectively from *G. parvifolia* leaves

The 45 L H *G. parvifolia* leaf extract showed the highest cytotoxicity towards Vero cells with CC_50_ of < 1.25 μg/mL and with cytotoxicity score of 1 (Fig. 1) followed by 45 L Ea and 45 L Et with CC_50_ of 237 and 555.0 μg/mL, respectively and both with cytotoxicity score of 3.

### Plaque reduction assay

**Fig. 2:**
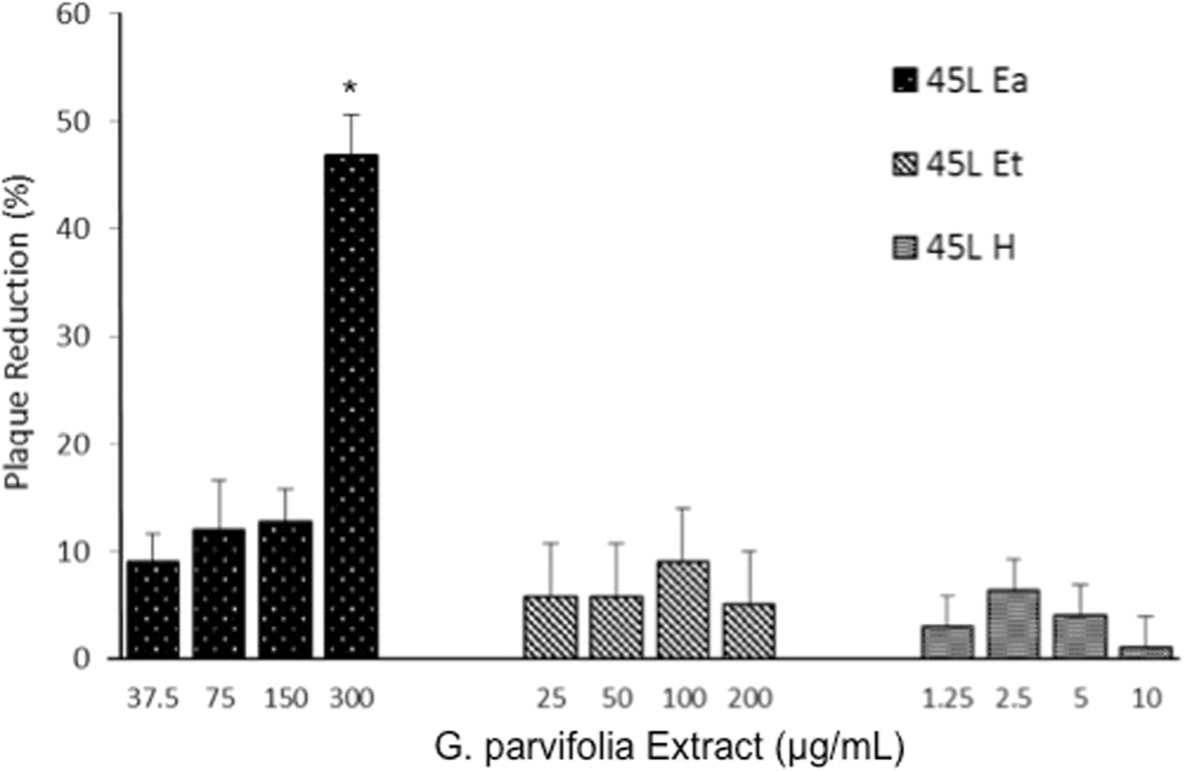
Effect of *G. parvifolia* extracts on Vero cell plaque reduction**.** Plaque inhibition is observed in cells treated with 45 L Ea only at a concentration-dependent manner. 45 L H, 45 L Et, and 45 L Ea are *G. parvifolia* hexane, ethanol, ethyl acetate leaf extracts, respectively. (*) indicates significant difference between individual extracts in each concentration at *p* < 0.05

There was significant (*p* < 0.05) dose-dependent Vero cell plaque reduction after treatment with 45 L Ea (Fig. 2).

### Viral inhibition

**Fig. 3:**
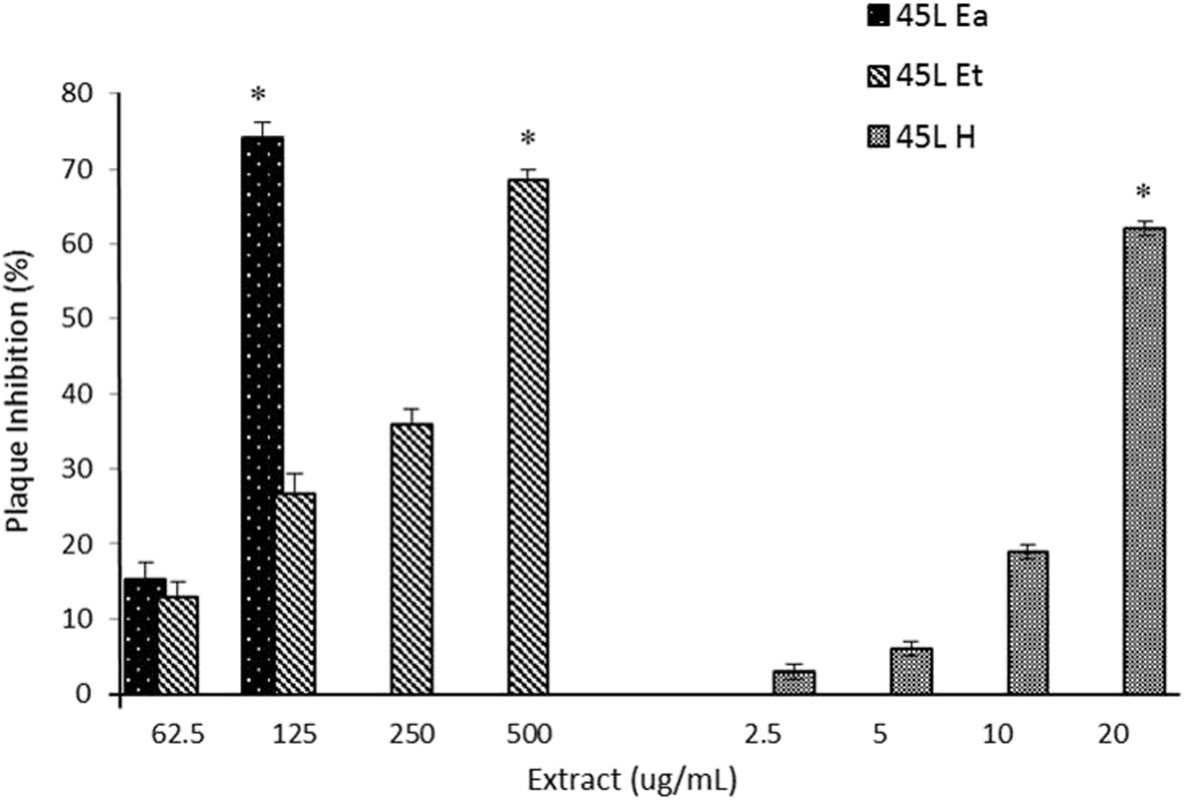
Effect of *G. parvifolia* extracts on Vero cell plaque inhibition**.** The greatest inhibitory effect was observed in the cell treated with 45 L Ea followed by 45 L Et. 45 L H, 45 L Et, and 45 L Ea are *G. parvifolia* hexane, ethanol and ethyl acetate leaf extracts, respectively. (*) indicates significant difference between individual extracts in each concentration at *p* < 0.05

All extracts irrespective of solvent used showed significant (*p* < 0.05) dose-dependent Vero cell plaque inhibition. However the CC_50_ concentration for 45 L H exceeded the safety level. Comparatively the 45 L Ea showed better inhibition (75%) of plaque formation than 45 L Et (26%) (Fig. 3).

### Viral attachment

**Fig. 4:**
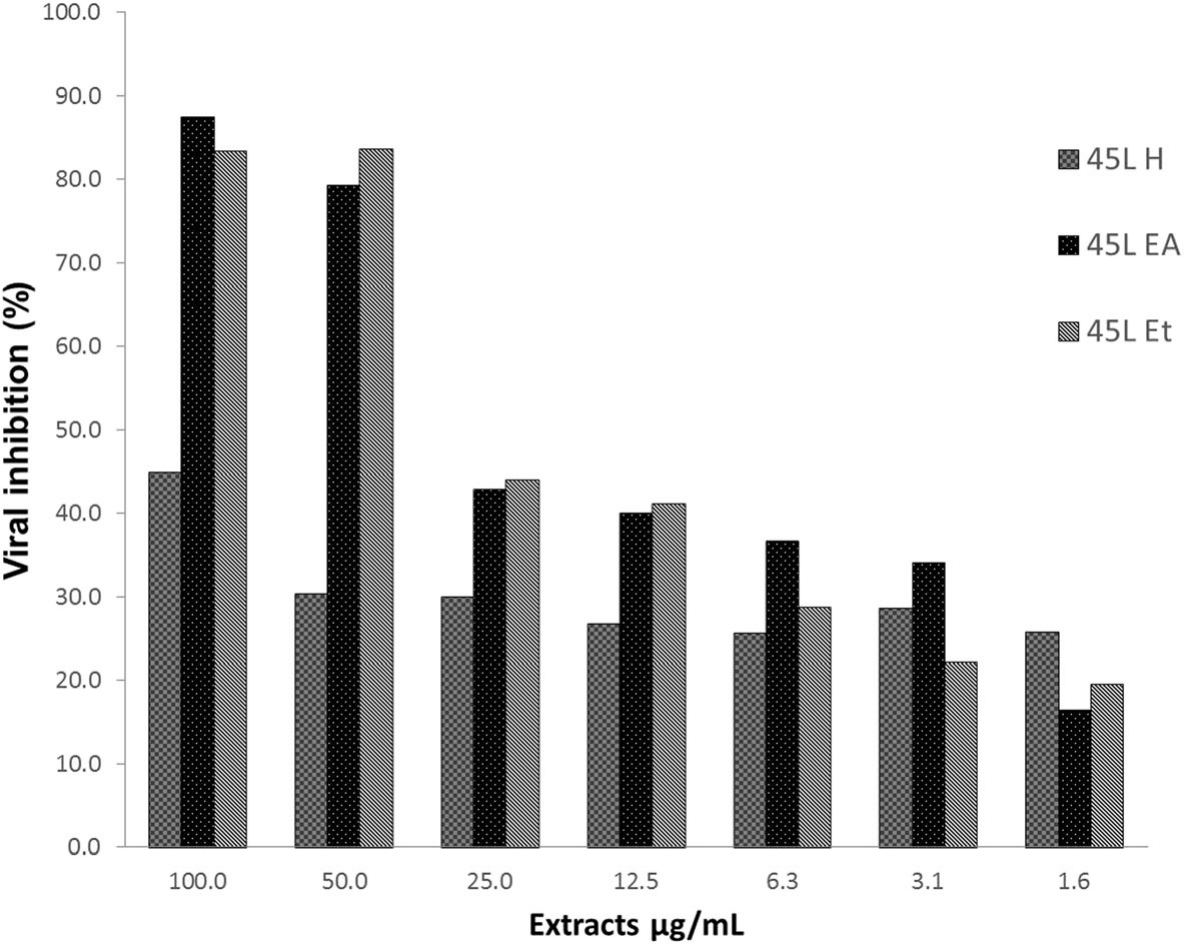
Effect of *G. parvifolia* extract on inhibition of viral attachment to Vero cells. 45 L H, 45 L Et, and 45 L Ea are *G. parvifolia* hexane, ethanol and ethyl acetate leaf extracts, respectively

Extracts, 45 L Ea and 45 l Et showed the greatest dose-dependent inhibition of viral attachment to Vero cells (Fig. 4). The 45 L H extract was not effective at inhibiting viral attachment to cells.

### Virucidal effect

**Fig. 5:**
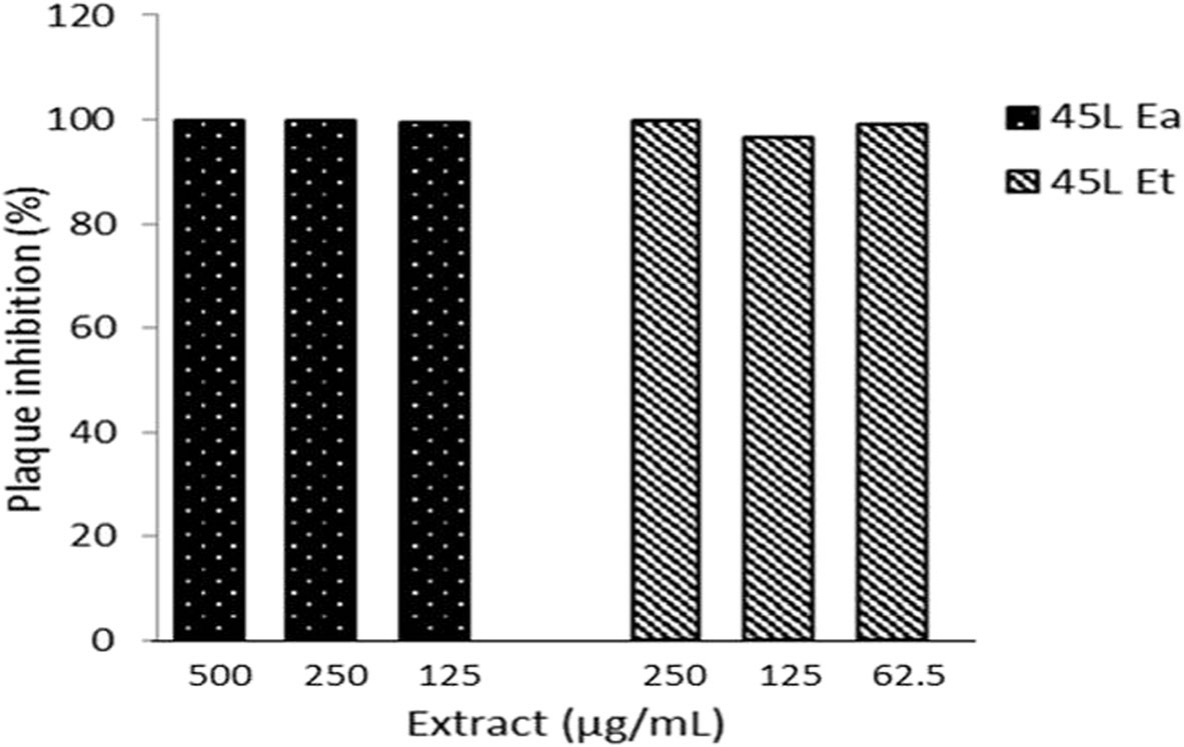
Virucidal activity of *G. parvifolia* extracts. The 45 L Ea and 45 L Et showed almost complete virucidal activity. 45 L H did not have any virucidal activity. 45 L H, 45 L Et, and 45 L Ea are *G. parvifolia* hexane, ethanol and ethyl acetate leaf extracts, respectively

The 45 L Ea and 45 L Et showed almost complete virucidal activity at all concentrations used in the study. The 45 L Ea had better antiviral effect than 45 L Et with residual infectivity of 1 and 4%, respectively (Fig. 5).

### Antiviral activity

The selectivity index (SI) [CC_50_/IC_50_] values for 45 L Ea, 45 L Et, and 45 L H were 2.65, 1.75, and 0.10, respectively. The SI showed that the 45 L Ea had the greatest antiviral activity followed y 45 L Et. The 45 L H had minimal antiviral activity.

## Discussion

This study focused on the three crude leaf extracts of *G. parvifolia*, namely 45 L H, 45 L Et, and 45 L Ea, which were extracted via ethyl acetate, ethanol and hexane solvents, respectively. The phytochemical constituents of these crude extracts were elucidated (Table 1). Based on the findings, 45 L Et contained all six phytochemical constituents tested and 45 L Ea contained five of them except saponin and the non-polar 45 L H did not contain saponin, tannin and phenolic contents. Among these, saponin and tannin are indeed very common secondary metabolites for plant kingdom, which possess good antioxidant activities. The presences of different classes of chemical constituents such as flavonoids, phenolics, terpenoids and steroids in 45 L are rather conformed to those isolated from different parts of *G. parvifolia* including leaves, twigs, latex, fruits and barks [8, 11]. In fact, flavonoids and terpenoids isolated from plants had been reported to contain antiviral properties, particularly against Chikungunya virus [28] and severe acute respiratory syndrome coronavirus [29].

**Table 1:** Phytochemical analysis on *Garcinia parvifolia* 45 L extracts

Sample	Type of Analysis					
	Saponin	Flavonoid	Tannin	Phenolic	Terpenoid	Steroid
45 L H	- (W)	- (D) yellow	- (W)	- (W)	+ (D) reddish brown	+ (D) yellow with green fluorescence
45 L Ea	- (W)	+ (D) pink	+ (W) white fog	+ (W) dark blue	+ (D) reddish brown	+ (D) yellow with green fluorescence
45 L Et	+ (W) frothing	+ (D) pink	+ (W) white fog	+ (W) dark blue	+ (D) reddish brown	+ (D) yellow with green fluorescence

- = constituent was not present in extract

+ = constituent was present in extract

(D) = extract was dissolved in DMSO solvent

(W) = extract was dissolved in purified water

Besides, further extraction and separation processes have yielded purer compounds from *G. parvifolia* as reported in previous studies. For instance, four novel prenylated depsidones had been isolated from the chloroform soluble fraction of the leaves of *G. parvifolia.* [30]. The leaf extracts also displayed the presence of a new benzoquinone derivative, namely parvifoliquinone and six other known compounds namely parvifoliol B, C (phloroglucinols), parvifoliol E (benzopyran derivatives), garcidepsidone B (depsidone), nigrolineaisoflavone A (isoflavone-like compound) and mangostinone (xanthone) [31].

Some depsidones have shown to be active against HIV by inhibiting the viral integrase [32]. Using molecular screening and docking investigations, certain Garcinia phytochemicals, including garcidepsidone had been reported to be potential inhibitors to inhibit the dengue viral replication inside the host cell with the help. Further in-vitro investigations require confirming their efficacy [33].

Clusianone had been isolated in abundance from the leaves of *G. parvifolia* through hexane extraction [34]. Recently, the anti-proliferative potential of structurally modified clusianone and its derivatives has been shown [35, 36]. A significant correlation was reported on the structure activity relationship of clusianone against Respiratory Carcinoma Cells via cytotoxicity assay [36]. The strong toxic effect of 45 L H extract towards Vero cells at minimal dose concentration, with CC50 of < 1.25 μg/mL, in comparison to 45 L Ea (CC50 of 237) and 45 L Et (555.0 μg/mL), could predict either a prominent cytotoxicity or the anti-proliferative effect of clusianone,

Previously, primary screening isolated 20 xanthones from plants of the Guttiferae family that have inhibitory effects on human herpesvirus 4 (HHV-4) also known as the Epstein-Barr virus (EBV). Xanthones, particularly 1,3,7-trihydroxy-2-(3-methyl-2-butenyl) xanthone, dulxanthone-B and latisxanthone-C, seemed to significantly inhibit EBA early antigen (EBV-EA), one of the viral genes required for the initiation complex at the lytic origin of viral replication in Raji cells [37]. Mangiferin with a broad spectrum beneficial biological activities, was the first xanthone shown to be pharmacologically effective for the treatment of diseases caused by herpesvirus, [38]. One of the effects of the xanthones is the inhibition of HIV-1 reverse transcriptase. Among xanthones, prenylated xanthones is restricted to the plant species of the family Guttiferae [38]. Prenylated xanthones namely mangostin and y-mangostin, isolated from *G. mangostana* are active against HIV-1 protease, preventing proteolytic cleavage during retroviral replication [38]. A recent investigation submitted a set of 272 xanthones to molecular docking examination and the results suggested that the xanthones could be suitable key components of agents to possess antiviral properties [39]. In fact, prenyl groups have been predicted to be important in protein-protein binding because of their specialized prenyl-binding domains that facilitate attachment to cell membranes. Therefore, a few lead compounds and their derivatizations could be screened using a structural-based docking method, or high-throughput screening methods for serving as a proof of concept for feasible viral target.

Although, there are a few studies that determined the antiviral effect of several *Garcinia* species [10, 40–42], reports were almost null on *G. parvifolia*. Owing to the limited antiviral research that has been done on this species, this study aims to enlighten researchers on the effect of *G. parvifolia* on PrV-infected Vero cells. It was shown that *G. parvifolia* ethyl acetate (45 L Ea) and ethanol (45 L Et) extracts particularly, did not only reduce but in fact almost completely inhibited Vero cell plaque formation. The antiviral effects of the extracts are proposed to occur through several mechanisms. The extracts inhibited viral infection of Vero cells as shown by reduction in plaque formation when PrV was firstly incubated with the extracts before exposure to Vero cells. The extracts also inhibited attachment of the virus to the cells. When the virus was allowed to infect Vero cells, the infected cells treated with *G. parvifolia* extract, plaque formation was inhibited. Similarly, when Vero cells were treated with extract-virus mixture, plaque formation was reduced. These effects were most prominent with 45 L Ea treatment. This finding suggests that the extract did not only inhibit cell infection by herpesviruses but also prevents viral replication of infected cells. The antiviral effect of *G. parvifolia* extracts are deemed to be virucidal. The virucidal property is only true for the ethyl acetate (45 L Ea) and ethanol (45 L Et) extracts, and not for the non-polar hexane (45 L H) extract. These more polar extracts caused almost total inhibition of plaque formation by the PrV-infected cells.

To realize the potential of a plant product as an antiviral compound, its mechanism of activity must be ascertained. It is important to differentiate between viral particle inactivation or virucidal activity from antiviral activity. Direct viral particle inactivation is an early effect where the virus is inactivated before it infects the cells while antiviral activity is the killing or suppression of replication ability of the virus. It would be ideal in virus infections for the treatment drug to possess both virucidal and antiviral activities. In our study, both the *G. parvifolia* ethyl acetate (45 L Ea) and ethanol (45 L Et) extracts presented with good virucidal activities of > 90%, and the effects of the extracts are partially on the inhibition of viral attachment and adsorption into target cells. The *G. parvifolia* hexane (45 L H) extract did not show similar activity on Vero cells.

Among approaches used in the determination of antiviral activity of natural compounds is the inhibition of viral DNA replication and reverse transcriptase. Most natural antiviral agents may act only on a limited number of viruses, because the viruses are prone to mutations that render the compounds eventually becoming ineffective [43, 44]. Antiviral compound should be highly effective while showing minimal toxicity to normal cells and tissues. One way determining potential of antiviral compound is by calculating the SI. In this study, the SI value of ethyl acetate (45 L Ea) extract was higher than either the ethanol (45 L Et) or hexane (45 L H) extract, indicating it has more potent antiviral activity. The results also suggest that the non-polar hexane (45 L H) extract was most toxic to Vero cells among the three extracts, thus may not be a suitable candidate as antiviral agent. The difference of phytochemical constituents between 45 L Ea (showing the highest antiviral potency) and 45 L H was the additional flavonoids, tannins and phenolics, which were extracted by ethyl acetate solvent. It might be possible that these constituents are responsible for the potent antiviral activities. It is worthy to mention that flavonoids, tannins, and phenolics had been found very important to inhibit different stages in the HIV replication cycle, where three of them act at the virus adsorption stage; flavonoids and tannins disable the reverse transcription and phenolics stop the viral integration in the human genome [32]. Besides, more recently, flavonoids of plant origin were found effective to combat against Chikungunya virus [28].

Based from a previous report on anti-bacterial screening, the hexane extract of the leaves of *G. parvifolia* did not show any antibacterial activity, while both hexane and ethyl acetate extracts of stem bark, root and fruit show strong antibacterial activity, especially against *Staphyloccocus aureus* [15]. Therefore, different parts of the plants possess diverse compounds and the extraction of compounds is influenced by the solvent used [45]. In this study, ethyl acetate was the best solvent for the bioactive extraction of *G. parvifolia* leaves, followed by ethanol; meanwhile, hexane did not seem to produce extracts with biological activity against PrV.

## Conclusion

The antiviral activity of the *G. parvifolia* extracts seemed to occur at several stages of the replication cycle. The multiple antiviral effects of the extracts are suggested to occur through the interference of viral attachment and traverse across cell membrane, cytoplasmic transport, and viral genome replication. Therefore, the antiviral effect of the *G. parvifolia* ethyl acetate (45 L Ea) and ethanol (45 L Et) extracts is suggested to occur through several mechanisms and not solely virucidal. This study suggests that the *G. parvifolia* extracts prevent viral replication in infected cells, particularly the 45 L Ea containing the flavonoids, tannins and phenolics which could be the constituents that are responsible for the potent antiviral activities. Therefore, future works will be emphasized on the pure compounds isolation from these 45 L Ea extract and to study the mechanisms of antiviral action triggered by these pure bioactives.

## Data Availability

The datasets used and/or analysed during the current study available from the corresponding author on reasonable request.

## Abbreviations

ATCC: American Type Culture Collection
CC_50_: The cytotoxic concentrations
CPE: Cytopathic effect
DMEM: Dulbecco modified Eagle medium
DMSO: Dimethyl sulfoxide
EA: Ethyl acetate
EC_50_: 50% effect concentration
ET: Ethanol
FBS: Fetal bovine serum
H: Hexane
IC_50_: 50% inhibitory concentration
MRSA: Methicillin-resistant *Staphylococcus aureus*
MTT: 3-(4,5-Diamethylthiazol-2-yl)-2,5-diphenyltetrazolium bromide
PBP2a: Penicillin-binding protein
PFU: Plaque forming unit
PrV: Pseudorabies virus
RPMI: Roswell Park Memorial Institute
SI: Selective index
TCID_50_: 50% tissue culture infective dose
